# 

**Published:** 1887-08

**Authors:** 


					﻿/olume LV.|
AUGUST, 1887.
[Number 2
^sssss
PEDICEL t
Journal?
AND
EXAMINER
EDITOR:
S. J. JONES, M.D., LL.D, 3
(LARK & LONGLEY (Q,(HI(AGO,
I	Publishers	I
				

